# Interoceptive brain network mechanisms of mindfulness-based training in healthy adolescents

**DOI:** 10.3389/fpsyg.2024.1410319

**Published:** 2024-08-13

**Authors:** Olga Tymofiyeva, Benjamin S. Sipes, Tracy Luks, Elissa J. Hamlat, Tara E. Samson, Thomas J. Hoffmann, David V. Glidden, Angela Jakary, Yi Li, Tiffany Ngan, Eva Henje, Tony T. Yang

**Affiliations:** ^1^Department of Radiology & Biomedical Imaging, University of California, San Francisco, San Francisco, CA, United States; ^2^Division of Child and Adolescent Psychiatry, Department of Psychiatry and Behavioral Sciences, Langley Porter Psychiatric Institute, University of California, San Francisco, San Francisco, CA, United States; ^3^Weill Institute for Neurosciences, University of California, San Francisco, San Francisco, CA, United States; ^4^Department of Epidemiology and Biostatistics, University of California, San Francisco, San Francisco, CA, United States; ^5^Department of Clinical Science/Child- and Adolescent Psychiatry, Umeå University, Umeå, Sweden

**Keywords:** adolescence, mindfulness, interoception, MRI, brain connectivity, mindfulness practices such as meditation

## Abstract

**Introduction:**

This study evaluated changes in the white matter of the brain and psychological health variables, resulting from a neuroscience-based mindfulness intervention, the Training for Awareness, Resilience, and Action (TARA), in a population of healthy adolescents.

**Methods:**

A total of 100 healthy adolescents (57 female, age ranges 14–18 years) were randomized into the 12-week TARA intervention or a waitlist-control group. All participants were imaged with diffusion MRI to quantify white matter connectivity between brain regions. Imaging occurred at baseline/randomization and after 12 weeks of baseline (pre- and post-intervention in the TARA group). We hypothesized that structural connectivity in the striatum and interoceptive networks would increase following the TARA intervention, and that, this increased connectivity would relate to psychological health metrics from the Strengths and Difficulties Questionnaire (SDQ) and the Insomnia Severity Index (ISI). The TARA intervention and all assessments, except for the MRIs, were fully remotely delivered using secure telehealth platforms and online electronic data capture systems.

**Results:**

The TARA intervention showed high consistency, tolerability, safety, recruitment, fidelity, adherence, and retention. After 12 weeks, the TARA group, but not controls, also demonstrated significantly improved sleep quality (*p* = 0.02), and changes in the right putamen node strength were related to this improved sleep quality (*r* = −0.42, *p* = 0.006). Similarly, the TARA group, but not controls, had significantly increased right insula node strength related to improved emotional well-being (*r* = −0.31, *p* = 0.04). Finally, we used the network-based statistics to identify a white matter interoception network that strengthened following TARA (*p* = 0.009).

**Discussion:**

These results suggest that the TARA mindfulness-based intervention in healthy adolescents is feasible and safe, and it may act to increase structural connectivity strength in interoceptive brain regions. Furthermore, these white matter changes are associated with improved adolescent sleep quality and emotional well-being. Our results suggest that TARA could be a promising fully remotely delivered intervention for improving psychological well-being in adolescents. As our findings suggest that TARA affects brain regions in healthy adolescents, which are also known to be altered during depression in adolescents, future studies will examine the effects of TARA on depressed adolescents.

**Clinical trial registration:**

https://clinicaltrials.gov/study/NCT04254796.

## Introduction

Young people show increased vulnerability to the development of mental illness, with an estimated 62.5% of individuals experiencing the onset of mental disorders before the age of 25 years (Solmi et al., 2022). Moreover, a recent meta-analysis including 80,879 young people globally showed that the prevalence of depression symptoms during COVID-19 has doubled, compared with pre-pandemic estimates, and the prevalence rates were higher when collected later in the pandemic (Racine et al., 2021). There is a great need for novel approaches to treat young people with current mental illnesses (e.g., depression and anxiety) and develop systems to promote resilience against mental illness.

Evidence suggests that depression and anxiety may be related to the interoceptive brain network (Terhaar et al., 2012; Harshaw, 2015), including in adolescents (Murphy et al., 2017). Specifically, some researchers believe that depression and anxiety disorders can cause people to misinterpret internal body signals (i.e., heartbeat or breathing sensations) to withdraw or avoid (Paulus and Stein, 2010). Increasing interoceptive awareness via strengthened connectivity of the interoceptive brain network may therefore allow more accurate interpretation of interoceptive signals and engagement with the world, especially in adolescents with depressive and/or anxious symptoms who struggle with this ability at the baseline. The association between the interoceptive brain network and positive mental health is further supported by a previous study which showed that the gray matter volume of the key interoceptive region, the insula, is positively associated with subjective well-being (Jung et al., 2022). This interoceptive functional brain network has been shown to be developed in adolescents and includes the insula, ACC, putamen, middle frontal gyri, middle temporal gyri, and middle occipital gyri (Klabunde et al., 2019). A previous study has shown that mindfulness as a practice improves well-being (Tang et al., 2021) and targets interoceptive brain regions (Farb et al., 2007; Gibson, 2019). Furthermore, rigorous clinical trials of mindfulness-based interventions in healthy adolescents have shown significant post-intervention reductions in symptoms of depression and anxiety as well as sleep disturbances (Kuyken et al., 2013; Blom Henje et al., 2016). These findings are reflected in large meta-analyses, which also indicate that mindfulness can significantly reduce depressive and anxious symptoms in adolescents (Chi et al., 2018; Zhou et al., 2020). Mindfulness practices generally involve closely attending to the body and breathing (Gibson, 2019), making them well-suited for strengthening interoceptive processes that involve similar mechanisms. In the brain, mindfulness practices involve the striatum (Tymofiyeva et al., 2022), as well as frontal and interoceptive regions in the ACC, insula, and dorsolateral prefrontal cortex (Farb et al., 2007; Gibson, 2019). Evidence suggests that mindfulness may remodel large-scale brain networks (Bremer et al., 2022) and reduce activation in the default mode network (Zhang et al., 2023). However, to the best of our knowledge, no randomized controlled clinical trial has studied the effectiveness of a mindfulness-based intervention on brain networks and the well-being of healthy (non-clinical) adolescents.

Our current study focuses on a promising approach to reducing depression in young people: a neuroscience-based mindfulness intervention, Training for Awareness, Resilience, and Action (TARA) that was initially conceptualized and designed to treat depression in adolescents by our group (Blom Henje et al., 2014). The theoretical framework of TARA is aligned with the Research Domain Criteria (RDoC) of the National Institute of Mental Health (NIMH) (Insel et al., 2010). Specifically, this fully manualized but semistructured 12-week group training, informed by mindfulness- and yoga-based techniques and modern psychotherapeutic approaches, has been designed to target the neurocircuitry involved in adolescent development and depression, including the striatum and interoceptive regions (Blom Henje et al., 2014). The striatum (the caudate and putamen) and interoceptive regions, such as the insula, have been previously shown to be engaged in contemplative practices (Lazar et al., 2000; Kjaer et al., 2002; Tang et al., 2009; Baerentsen et al., 2010; Sharp et al., 2018). This study not only focuses on the exhaustive list of the regions and circuits that TARA may influence but also primarily centers on them. We intend to investigate other targets described in the concept study by Blom Henje et al. (2014) in future studies.

A preliminary trial of TARA showed significant improvements in depression, anxiety, and sleep in depressed adolescents following the TARA intervention (Blom Henje et al., 2016). We also observed an improvement in emotional well-being in healthy adolescents after 12 weeks of TARA (Blom Henje et al., 2016). Specifically, a significant decrease in anxiety symptoms with TARA was observed in healthy adolescents compared with a control time period (Tymofiyeva et al., 2021), as well as changes in structural brain connectivity (Tymofiyeva et al., 2021) and gray matter volume (Yuan et al., 2020), including the striatum. While Tymofiyeva et al. (2021) and Yuan et al. (2020) used a within-subject design where subjects served as their own controls, the current study uses a between-subject randomized controlled trial (RCT) design, which compares teens who receive TARA to separate waitlist controls in a unique sample of healthy adolescents, to understand whether TARA leads to increased white matter connectivity in striatal and interoceptive regions and whether changes in these brain systems are associated with improved psychological functioning when compared with waitlist controls. Our focus on white matter connectivity is due to several reasons: (1) white matter (structural) connectivity reflects relatively fixed anatomic connections in the brain, upon which all transitory functional states unfold (Sporns and Tononi, 2007); (2) white matter connectivity is compromised in mental disorders, including adult and adolescent depression (Korgaonkar et al., 2020; Tymofiyeva et al., 2017); (3) optogenetic animal studies show that white matter (myelin) remodeling causally underlies improvement with training (Gibson et al., 2014), and finally, (4) our preliminary study in adolescents (Tymofiyeva et al., 2021) and other researchers’ studies in adults (Sharp et al., 2018) showed changes in node strength with 12–15 weeks of contemplative practices.

The current study conducts a single-blind randomized controlled trial with the fully remotely delivered TARA mindfulness-based intervention in healthy adolescents. We assessed whether the striatum and the adolescent interoceptive network show changes in structural connectivity following the TARA intervention. Based on our published study (Tymofiyeva et al., 2022), our primary hypothesis was that the left putamen structural connectivity strength would increase more in the TARA intervention group compared with the waitlist control group. To ensure a comprehensive analysis, we also explored changes in regional connectivity strength in the bilateral putamen, caudate, and two key regions in the interoceptive network, the ACC and insula, between the two groups. Our secondary hypothesis was that changes in structural connectivity strength would be related to improved sleep quality and emotional well-being. To enhance the interpretability of both analyses, we used the network-based statistic to assess whether the adolescent interoceptive network increased in connectivity strength following the 12-week TARA intervention.

## Materials and methods

The study received approval from the Institutional Review Board (IRB) of the University of California, San Francisco. Informed assent and consent were obtained from the study participants and their parent(s) or legal guardian(s), respectively, in accordance with the principles outlined in the Declaration of Helsinki. The original study was registered on ClinicalTrials.gov (NCT04254796).

### Participants and experimental design

Healthy male and female adolescent participants were recruited using flyers in San Francisco high schools, nearby areas, and through online. Inclusion criteria were that participants had to be between the ages of 14 and 18 years old, fluent in English, free of any self- or parent-reported medical or psychiatric illnesses at baseline, and had access to a device on which Zoom could be installed. Participants were excluded if they were taking psychotropic medications, had MRI contraindications, were pregnant, or were already practicing mindfulness for at least 20 min twice or more per week, which minimized the potential for previous mindfulness experience to confound the results.

**Figure 1:**
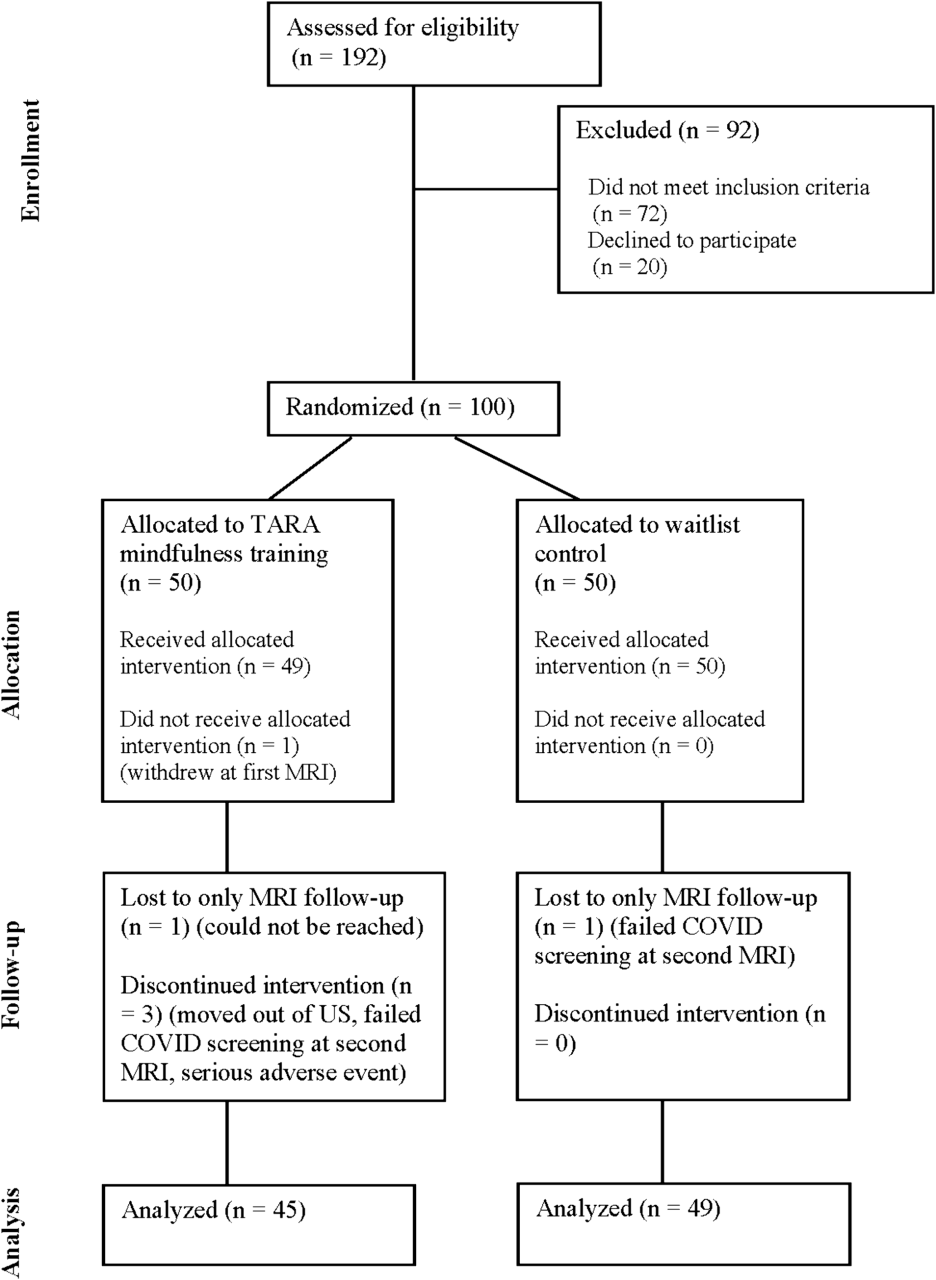
CONSORT diagram.

The study was an individually randomized, open-label, waitlist-controlled clinical trial. At the baseline, 100 healthy female (57) and male (43) adolescents, mean age of 15.93 (1.33) years, were block randomized to the TARA intervention group or the waitlist control group. MRI assessments of the brain’s structural connectivity and fully remote assessments of emotional symptoms occurred at baseline and 12 weeks after baseline (pre- and post-intervention for the training group) (see Figure 1 CONSORT diagram). The principal investigators, MRI assessors, and clinical assessors were blinded to the group assignment.

Participants received up to $220 in gift cards for the completion of the study.

### TARA intervention

As described in the introduction, Training for Awareness, Resilience, and Action (TARA) (Blom Henje et al., 2014) is a structured 12-week group intervention for adolescent depression and takes into account the developmental aspects of the adolescent brain. TARA incorporates mindfulness-based practices, including breathing techniques, yoga-based movements, and guided meditation such as interoceptive/sensory awareness practices called “body-scans,” psychoeducation, and practices in which the suffering of depressive symptoms is contextualized and addressed from a systematic perspective. The psychoeducation presentations were designed to provide the rationale for each of the practices, and topics included stress responses, the functioning of the autonomous nervous system, and strategies to improve emotion regulation. TARA consists of four modules (Blom Henje et al., 2014, 2016): the first module (weeks 1–3) aims to promote vagal and sensory activation through breathing practices and slow synchronized movement, which is expected to decrease feelings of stress and physical symptoms of depression and anxiety in addition to improved emotional self-regulatory abilities and sleep. The second module (weeks 4–6) involves noticing negative self-referential processing and then shifting to present moment sensory and interoceptive awareness. The third module (weeks 7–9) aims to bring the acquired skill set into managing emotions during social interactions through recognizing emotional triggers in oneself and others, empathetic listening, effective communication, and compassionate responses to oneself and others, which is expected to decrease social anxiety. The fourth and last module (weeks 10–12) targets the striatal brain system and aims to increase behavioral activation guided by intrinsic reward, inspired by techniques in acceptance and commitment therapy, which is expected to decrease experiential avoidance, increase committed action, and improve behavioral effectiveness amidst distressing emotional experiences.

A total of 90 min weekly sessions were delivered by two TARA-trained facilitators fully remotely over Zoom for 12 consecutive weeks—18 h in total. The suggested weekly home practice could last anywhere from 5 min to 1 h. To facilitate home practice, audio recordings were provided to the participants, containing guided meditations, yoga-based movement sequences, and breathing exercises. More details are provided in the conceptual and feasibility studies (Blom Henje et al., 2014, 2016). The TARA facilitators were all trained to teach TARA by the lead TARA developer and author EH. Before teaching TARA, they were all asked to sit in on one full TARA course and then co-teach with a facilitator with many years of experience. While the feasibility of remote delivery of TARA over Zoom has been demonstrated (Tymofiyeva et al., 2022), our previous study was limited by the small sample size and the fact that the second half of the 12-week intervention was delivered remotely. Therefore, one goal of the present study was to assess the feasibility of a fully remotely delivered TARA intervention. The fidelity and consistency of the intervention were monitored and scored fully remotely by trained research staff members. See the Supplementary material for more details.

Treatment safety was monitored and measured fully remotely as the number of adverse events in TARA participants was captured as follows: (1) Before each TARA session, participants were asked about the Child Outcome Rating Scale (CORS) “Has there been any significant unfavorable change in your mental or physical health since the last class?”; (2) If the participant answered “yes” to this question, then a study staff member asked the participant for additional details regarding the participant’s answer including whether the unfavorable change was related to the TARA intervention, and this information was recorded in the adverse event form.

Treatment fidelity and consistency were also monitored fully remotely. To monitor treatment fidelity and consistency, we used the 10-item fidelity scale (Clarke, 1998), facilitator self-monitoring, and monitored patient adherence. All group sessions were audio-recorded; the research consent process included consent to audio-recorded groups to facilitate treatment fidelity coding. A random sample (20%) of group sessions was rated for adherence to the training manual and facilitator competence using the 10-item fidelity scale (Clarke, 1998). Training facilitators completed self-monitoring of adherence to each session by completing a checklist of specific practices after each group. Additionally, we monitored participant adherence (e.g., attendance and home practice completion) and non-specific adherence information (e.g., session cancelation, waiting time) using a fact sheet completed by the training facilitators after each group.

The participants’ acceptability and tolerability of TARA were assessed weekly using the Child Session Rating Scale (CSRS) (Campbell and Hemsley, 2011). The CSRS is a 4-item self-assessment using a 10-cm visual analog scale, with higher scores indicating better experience with the intervention. Participants rated each session in terms of how much they felt listened to (using a continuous scale between “The teachers did not always listen to me” and “The teachers listened to me”), how important the content and activities were to them (using a continuous scale between “What we did and talked about was not really that important to me” and “What we did and talked about were important to me”), how much they liked the session (using a continuous scale between “I did not like what we did today” and “I liked what we did today”), and their overall experience (choosing on a continuous scale between “I wish we could do something different” and “I hope we do the same kind of things next time”).

### MRI data acquisition and preprocessing

MRI scanning was performed on a 3 T GE Discovery MR750 (General Electric Healthcare, Milwaukee, WI, USA) with a Nova 32-channel head coil (Nova Medical, Wilmington, MA, USA). The scan included a standard high-resolution T1-weighted scan for the purposes of anatomical alignment and identification of the nodes of the MRI connectome and a diffusion-weighted MRI scan with a spinecho echo-planar-imaging (EPI) sequence (TR = 7.5 s, minimum TE, FOV = 25.6×25.6 cm, 128×128 matrix, slice thickness = 2 mm, *b*-value of 1000s/mm^2^, 30 non-collinear directions of diffusion sensitizing gradients, and one b_0_ volume) for the purposes of conducting fiber tractography and deriving connections/edges of the MRI connectome.

MRI data preprocessing was done using the FMRIB Software Library (FSL) (Smith et al., 2004) and in-house scripts in MATLAB (MathWorks, Natick, MA). Diffusion volumes affected by motion were rejected in a quality assurance step (Tymofiyeva et al., 2012), and the remaining images were corrected for eddy current distortions, affine head motion, and b-vector rotation to account for head motion. Diffusion Toolkit was used to perform Diffusion Tensor Imaging (DTI) reconstruction, deterministic whole brain streamline fiber tractography, and visualization (Wang et al., 2007). Fiber Assignment by Continuous Tracking (FACT) was conducted with one seed per voxel and a standard threshold angle of 35°.

### MRI connectome construction

Brain segmentation, including the cerebellum, into 116 nodes/regions of interest (ROIs) was performed in DTI space using the Automated Anatomical Labeling (AAL) atlas (Tzourio-Mazoyer et al., 2002) via intermediate registration to T1-weighted images in MNI space. The connectome edges were calculated by using weights as the average fractional anisotropy (FA) sampled along the connecting streamlines, which has been shown to produce reliable and highly precise graph metrics in adolescents (Yuan et al., 2019). The resulting MRI connectomes were represented by 116 × 116 connectivity matrices, in which each row/column corresponds to a distinct node (brain ROI), and each entry corresponds to an FA-weighted edge/connection.

### Psychological health self-assessment measures

Our secondary outcome measures included two psychological health variables. For assessing emotional well-being, we used the emotional symptoms subscale of the Strengths and Difficulties Questionnaire (SDQ) (Goodman and Goodman, 2009), which has good psychometric properties and is widely used to evaluate adolescent mental health (Goodman, 2001). Adolescents with elevated SDQ scores are more likely to concurrently and prospectively (3 years later) experience clinical-level mental health disorders, including depression (Goodman and Goodman, 2009). The emotional subscale of the SDQ has been shown to have a very good ability to differentiate cases of anxiety disorders and/ or depression from non-cases (Blom Henje et al., 2010). The SDQ asks about emotional experiences over the past 6 months. The emotional symptom subscale items include “I worry a lot” and “I am often unhappy, depressed, or tearful.” Scores on the subscale range from 0 to 10, with higher scores indicating greater emotional problems. Insomnia Severity Index (ISI) measuring sleep disturbances was also a secondary measure. The ISI is a brief 7-item assessment scale for insomnia (Chung et al., 2011) with scores ranging from 0 to 28. All psychological health measures including the SDQ and ISI were collected fully remotely and securely through Qualtrics for both baseline and follow-up assessments. For more details on how recruitment, intervention, and assessment were completed, see the Supplementary materials.

### Statistical analyses

While there are many ways to measure structural connectivity differences, one approach we have used previously is to quantify the overall connectivity strength in individual regions (Tymofiyeva et al., 2022), where node strengths were defined as the sum of connection weights between the node of interest and all other nodes in the network (Rubinov and Sporns, 2010).

We hypothesized that structural connectivity node strength in adolescents in the intervention group would increase more (or decrease less) than in the waitlist control group. Our primary hypothesis was that the left Putamen node strength would increase following TARA. However, to be more comprehensive, we further explored whether structural connectivity strength changed in striate and key interoceptive regions such as the bilateral Putamen, Caudate, Insula, and Anterior Cingulate Cortex (ACC) using the AAL atlas regions of interest. These regions were chosen based on their theoretical involvement in the TARA intervention and their relationship with mindfulness practice (Blom Henje et al., 2014; Gibson, 2019). We also hypothesized that the SDQ emotional subscale and ISI for adolescents in the intervention group would improve more (or show less deterioration) than in the waitlist control group and that changes in these outcome variables would relate to changes in regional connectivity strengths. Due to the relatively small sample size and exploratory nature of this investigation, we did not correct for multiple comparisons for these analyses.

Before testing these hypotheses, outlier analysis on structural connectivity values was performed using Tukey’s method (Tukey, 1977), and cases of outcome variable changes with values >3 times the interquartile range (IQR) were removed.

Given that there are sex- and age-related differences in psychological functioning during adolescence, particularly in depression (Nolen-Hoeksema and Girgus, 1994; Frey et al., 2020; Hosozawa et al., 2024), and as with most research in this area, we used sex and age as covariates in our analyses. Physical development during adolescence is also reflective of sex and age; this appears to include brain development and connectivity, as evidence emerges in this area (Dennis et al., 2013; Ingalhalikar et al., 2014; Lim et al., 2015; Kaczkurkin et al., 2019). Research examining structural connectivity in adolescents often accounts for both sex and age, so we followed this convention in our subsequent analyses.

To test for the effects of TARA and waitlist control on structural connectivity and psychological health, changes (difference scores) were calculated as the difference in pre- and post-intervention variable values. We performed Analyses of Covariance (ANCOVA) on the changes (difference scores) as outcome variables, with a group (TARA or waitlist control) as a between-subject factor, controlling for mean-centered age and biological sex. We applied Bonferroni corrections for multiple comparisons separately for the 2 psychological health outcomes and the 8 structural connectivity outcomes. To explore group differences in the relationships between changes in structural connectivity and changes in psychological health, we performed ANCOVA on the changes as outcome variables, with a group (TARA or waitlist control) and change (difference score) in node strength as between-subject factors, controlling for mean-centered age and sex. As a *post-hoc* analysis to further examine the within-group relationship between node strength and psychological health for significant group interaction results in the ANCOVA analyses, we computed Pearson’s correlation within each group (with no correction for multiple comparisons). These statistical analyses were performed using JMP SAS software (version 17pro). As a sensitivity test for the effects of baseline scores on the ISI and SDQ, we ran a linear mixed model with covariates similar to the previous model, but additionally including pre/post time, an interaction between pre/post time and group (the coefficient of interest), and a random effect for individual, using the R v4.1.0 (R Core Team, 2021) package lme4 v1.1.32 (Bates et al., 2015).

### Network-based statistic

We further used the network-based statistic (NBS; Zalesky et al., 2010) as an inductive approach to assess white matter network changes following the 12-week TARA intervention. NBS attempts to find a subnetwork of connectivity across multiple regions related to an outcome measure while correcting for multiple comparisons (Beare et al., 2017; Sipes et al., 2022). We performed this exploratory NBS analysis using the regions hypothesized to be targeted by the TARA intervention combined with those identified recently as related to adolescent interoceptive function (Klabunde et al., 2019). In addition to the above target regions, this network includes structures around the brain including the frontal gyri, middle occipital gyri, and middle temporal gyri, which were shown to have increased fMRI BOLD signal during an interoceptive heartbeat attention task (Klabunde et al., 2019). We entered the FA-weighted SC change matrices into network-based statistic (NBS) software (MATLAB version NBS1.2) along with age and sex as controlled covariates. In NBS, each edge is tested independently and assigned a test statistic, and then, a subnetwork is identified based on a threshold for this test statistic. The statistical significance of this subnetwork is corrected for multiple comparisons and is based on comparing the empirical subnetwork’s size to a null distribution of 10,000 randomized networks. Selecting an appropriate test statistic threshold is crucial since a lower threshold indicates an overall weaker effect, and more randomized networks are likely to also be significant, while a higher threshold indicates a stronger effect but may not form a connected subnetwork across regions. Given the exploratory nature of this analysis and the fact that we did not have strong *a priori* expectations for the effect size, we tuned the test-statistic threshold across multiple thresholds from 1 to 2, and we chose a test statistic that produced a statistically significant subnetwork based on the subnetwork’s component size (i.e., extent).

## Results

### TARA intervention recruitment, retention, consistency, tolerability, and safety

We successfully recruited and accrued 100 healthy adolescents to participate in the study via fully remote methods that met data security standards for collecting and assessing individual-level data. All participants were enrolled and randomized in the study using secure remote methods, meeting the target recruitment number (*n* = 100) for this clinical trial. A total of 50 participants were randomized into the TARA group and 50 into the waitlist control group (Table 1). There were 28 female and 22 male adolescents in the TARA group and 29 female and 21 male adolescents in the control group (between groups chi-square = 0.063, *p* = 0.8). The average age was 16 years in both groups: TARA: mean = 15.8 (1.4; 14.0–19.0), controls: mean = 16.0 (1.3; 14.0–18.8) (between groups *t* = 1.0, *p* = 0.32).

**Table 1:** Demographic and baseline characteristics of the study participants.

	TARA group	Control group	Group difference test	*p*
Number of participants (baseline)	50	50		
Number of participants (completed study)	45	49	Chi Square = 1.73	0.19
Biological sex (female/male)	28/22	29/21	Chi Square = 0.063	0.8
Mean age at baseline (st dev; range)	15.8 (1.4; 14.0–19.0)	16.0 (1.3; 14.0–18.8)	*t* = 1.0	0.32
Race			Chi Square = 5.82	0.21
White/Caucasian	25 (50%)	28 (56%)		
African American	1 (2%)	3 (6%)		
Asian	5 (10%)	9 (18%)		
Native American	0 (0%)	0 (0%)		
Pacific Islander	0 (0%)	1 (2%)		
Other or more than one	19 (38%)	9 (18%)		
Ethnicity			Chi Square = 2.75	0.25
Hispanic or Latino	11 (22%)	5 (10%)		
Not Hispanic or Latino	38 (76%)	44 (88%)		
Unknown	1 (2%)	1 (2%)		
Socio-economic status/the highest level of education of the mother			Chi Square = 3.21	0.67
Less than 9th grade	0 (0%)	0 (0%)		
Some high school	4 (8%)	3 (6%)		
Graduated high school	3 (6%)	6 (12%)		
Some college	5 (10%)	2 (4%)		
Graduated college	16 (32%)	14 (28%)		
Professional/graduate degree	22 (44%)	24 (48%)		
Do not know	0 (0%)	1 (2%)		
Handedness			Chi Square = 1.11	0.29
Right	45 (90%)	45 (90%)		
Left	5 (10%)	5 (10%)		
Mean baseline Insomnia Severity Index (st dev; range)	7.86 (5.06; 0–18)	4.57 (3.98; 0–15)	*t* = 13.01	0.0009
Mean post-intervention Insomnia Severity Index (st dev; range)	6.86 (0.78; 0–20)	5.26 (4.76; 0–21)	*t* = −1.52	0.13
Mean baseline SDQ emotional problems (st dev; range)	52.46 (11.29; 37–84)	50.66 (10.26; 37–75)	*t* = −0.81	0.42
Mean post-intervention SDQ emotional problems (st dev; range)	54.74 (12.31; 37–84)	50.85 (10.83; 37–80)	*t* = −1.61	0.11

In total, 45 participants in the TARA group and 49 participants in the control group completed the study. All of these participants completed pre- and post-intervention MRIs. One of the participants (control) did not fill out the post-intervention SDQ emotional subscale, and three participants (two controls and one TARA participant) did not fill out post-intervention ISI. After dropouts, the final sample included 94 subjects; of which, 45 subjects undergoing TARA and 49 were waitlist controls. Therefore, the clinical trial achieved a 94% retention rate using fully remote methods. One SDQ emotional subscale change outlier was removed.

The TARA intervention was delivered consistently: 94% of subjects participated in at least 75% of sessions, demonstrating good adherence to the intervention. Participants enrolled in TARA reported strongly positive session experiences on the Child Session Rating Scale (CSRS): 100% of TARA participants had median CSRS scores of >20, indicating that TARA was well tolerated. There were zero intervention-related adverse events, indicating that TARA was exceptionally safe and well-tolerated. For more details on recruitment, retention, consistency, tolerability, and safety, see the Supplementary material.

### Effects of TARA on changes in psychological health and self-assessment measures

**Figure 2:**
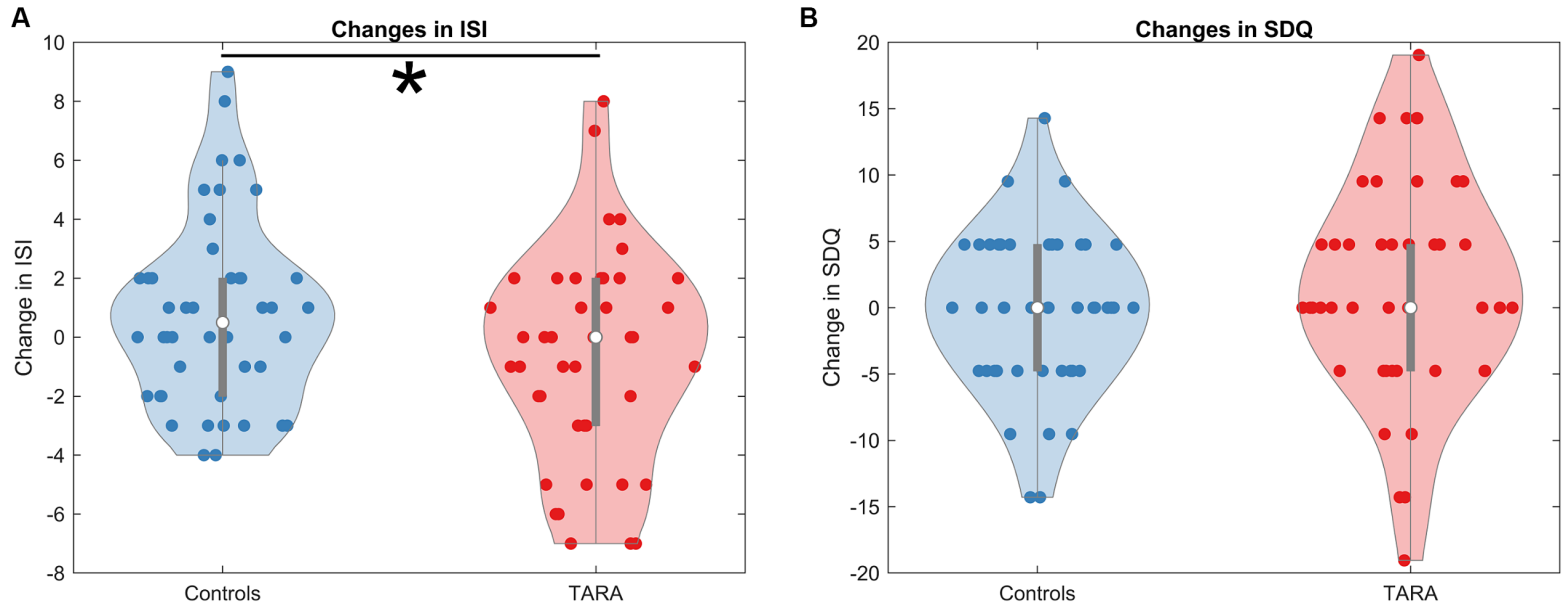
Changes in ISI and SDQ. We show the changes in **(A)** the Insomnia Severity Index (ISI) and **(B)** the Strengths and Difficulties Questionnaire (SDQ--emotional problems subscale). The ISI shows a significant reduction in the TARA group, indicating that the TARA mindfulness intervention significantly improved sleep quality in adolescents. The change in SDQ was not significant.

The TARA group had a significantly larger decrease in ISI scores (improved sleep quality) than controls (controlling for age and sex, *F* = 5.54, *p* = 0.021, significant with Bonferroni corrected *p*-value threshold of 0.025) (Table 2; Figure 2); the result remained significant in a sensitivity analysis incorporating baseline in a linear mixed model (*p* = 0.021). There were no significant differences between groups for changes in SDQ Emotional Problems scores; the results were similar after a similar sensitivity analysis incorporating baseline in a linear mixed model (*p* = 0.31). There was no correlation between changes in ISI and SDQ scores across groups (*r* = 0.11, *p* = 0.41) or within the waitlist control group (r = 0.05, *p* = 0.76) or the TARA group (*r* = 0.15, *p* = 0.34).

**Table 2:** Effects of TARA on changes in psychological health measures and structural connectivity, controlling for age and sex.

	TARA group Mean (st dev)	Waitlist Control group Mean (st dev)	*F*	*p*	Cohen’s d
Change in SDQ emotional problems score	1.44 (8.3)	−0.405 (5.9)	1.63	0.205	0.168
Change in Insomnia Severity Index score	−0.74 (5.1)	0.74 (4.0)	5.54	**0.0209**	**0.445**
Change in left putamen node strength	1.12 (4.6)	−0.08 (5.5)	1.43	0.234	0.25
Change in right putamen node strength	−0.34 (3.9)	−0.1 (4.2)	0.014	0.905	0.058
Change in left insula node strength	0.27 (3.6)	0.30 (3.6)	0.004	0.948	0.008
Change in right insula node strength	0.38 (3.8)	−0.22 (3.8)	0.913	0.342	0.157
Change in left caudate node strength	0.57 (4.7)	−0.72 (5.6)	0.113	0.233	0.252
Change in right caudate node strength	1.31 (4.7)	−1.0 (5.2)	5.07	**0.027**	**0.470**
Change in left ACC node strength	0.03 (3.8)	−0.47 (4.3)	0.314	0.577	0.11
Change in right ACC node strength	0.21 (2.6)	−0.22 (4.4)	0.318	0.574	0.12

Changes were calculated as the difference in pre- and post-intervention variable values. Bold indicates *p*-values less than 0.05.

### Effects of TARA on changes in structural connectivity

For our primary outcome measure, we found that the left putamen node strength increased by the predicted effect size (Cohen’s d = 0.23), but that this effect was not significant (*F* = 1.43, *p* = 0.23).

**Figure 3:**
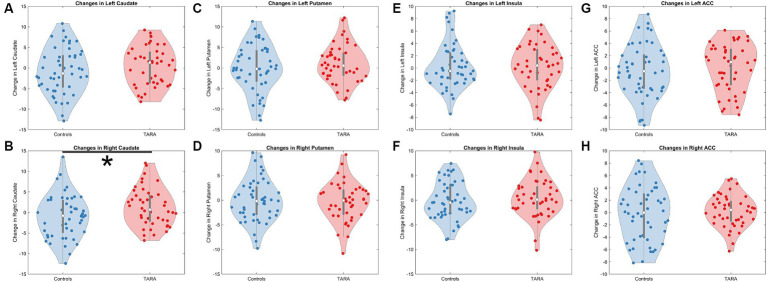
Changes in structural connectivity node strength. We show violin plots of all evaluated regions, which included the **(A)** left caudate, **(B)** right caudate, **(C)** left putamen, **(D)** right putamen, **(E)** left insula, **(F)** right insula, **(G)** left anterior cingulate cortex (ACC), and **(H)** right ACC. Only the right caudate **(B)** showed a significant increase in node strength following TARA.

For the exploratory analysis with other regions, we observed a significant increase in the right caudate node strength in TARA compared to controls (*F* = 5.54, *p* = 0.027, not significant with Bonferroni correction for multiple comparisons) (Table 2; Figure 3). There were no other nodes with significant group differences in changes in node strength.

### Effects of TARA on the relationship between changes in structural connectivity and changes in psychological health

**Figure 4:**
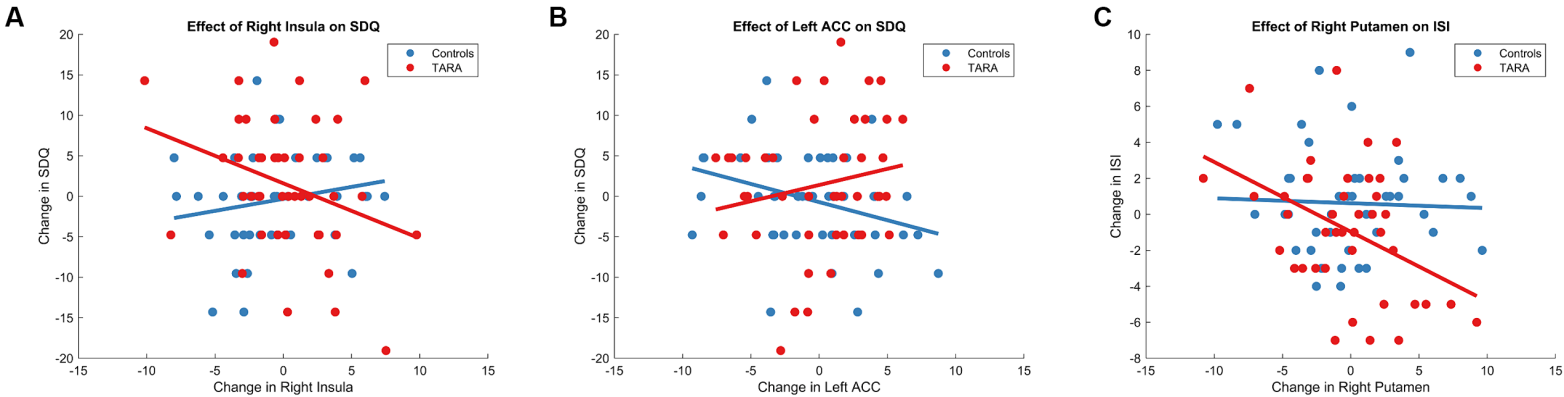
TARA effect of regions on sleep and emotional well-being. We show the significant group-level effects between the change in SDQ and **(A)** the right insula and **(B)** the left ACC. We also show the significant group-level effect between the changes in **(C)** the ISI and right putamen.

Aligned with this hypothesis, we found that there was a difference between groups in the relationship between changes in right insula node strength and SDQ Emotional Problem scores (controlling for age and sex, *F* = 5.43, *p* = 0.022). In the TARA group, increases in right insula node strength were associated with decreases in SDQ Emotional Problem scores (*r* = −0.31, *p* = 0.04). In the waitlist control group, there was no significant relationship between changes in right insula node strength and SDQ Emotional Problem scores (*r* = 0.19, *p* = 0.2) (Figure 4). No results were significant after Bonferroni corrections.

We similarly found that there was a difference between groups in the relationship between changes in left ACC node strength and SDQ Emotional Problem scores (controlling for age and sex, *F* = 5.64, *p* = 0.0198) (Figure 4). In the TARA group, there was no significant relationship between changes in left ACC node strength and SDQ Emotional Problem scores (*r* = −0.17, *p* = 0.11). In the waitlist control group, increases in left ACC node strength were associated with decreases in SDQ Emotional Problem scores (*r* = −0.32, *p* = 0.03). No results were significant after Bonferroni corrections.

For sleep quality, we found that there was a trending difference between groups in the relationship between changes in right putamen node strength and Insomnia Severity Index scores (controlling for age and sex, *F* = 3.8410, *p* = 0.0535) (Figure 4). In the TARA group, increases in right putamen node strength were associated with decreases in Insomnia Severity Index scores (*r* = 0.42, *p* = 0.006). In the waitlist control group, there was no significant relationship between changes in right putamen node strength and Insomnia Severity Index scores (*r* = 0.075, *p* = 0.62). No results were significant after Bonferroni corrections.

There were no other significant differences between groups (or across groups) in the relationships between changes in node strengths and Insomnia Severity Index scores or SDQ Emotional Problem scores.

### Adolescent interoception network changes with TARA

**Figure 5:**
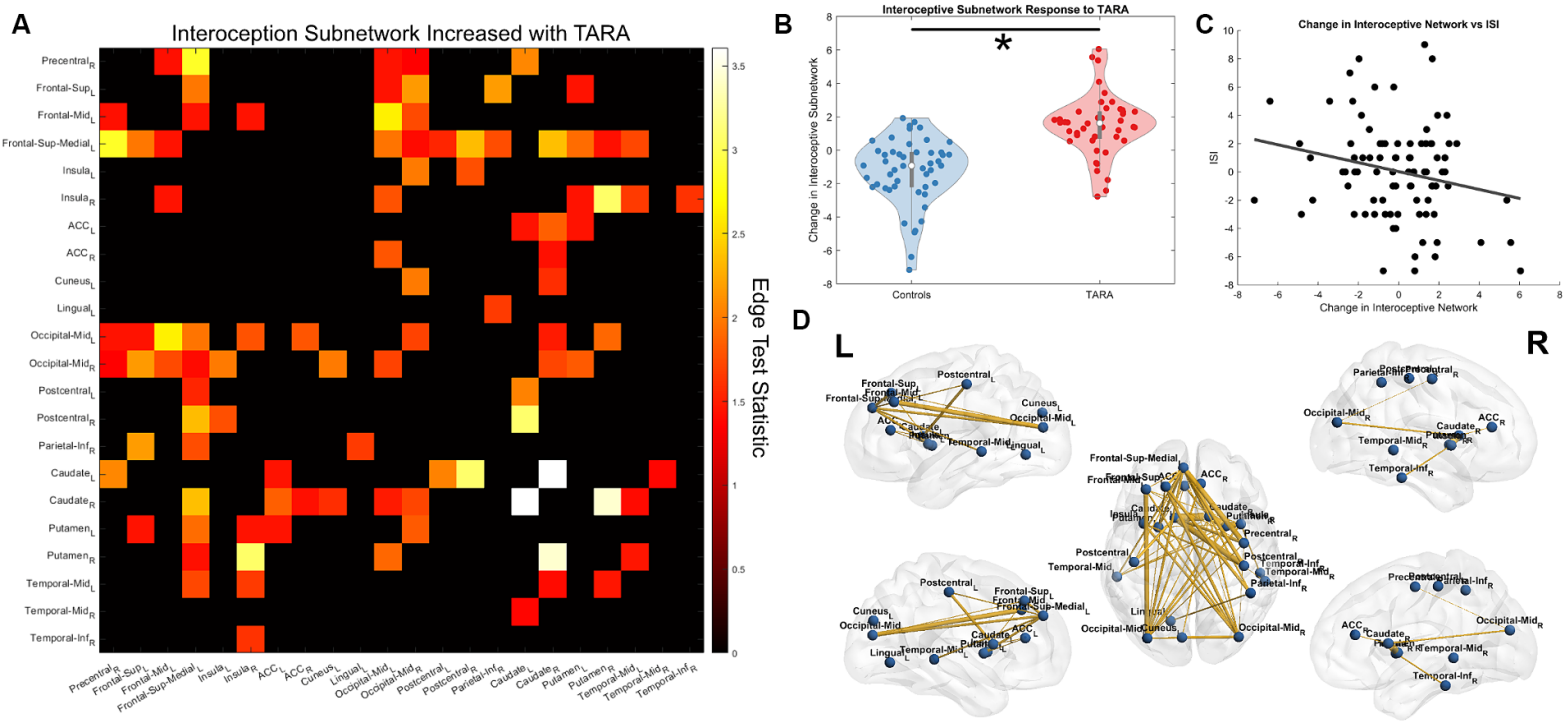
Interoception network strengthening from TARA. **(A)** We show the significant subnetwork identified with the network-based statistic (NBS). Each row/column is labeled with the according ROI. **(B)** The total change in the subnetwork (sum of all edge changes) reflecting the significant difference identified by NBS. **(C)** The *post-hoc* analysis finding a significant correlation between the interoception subnetwork change across all study subjects and improved sleep quality (i.e., decreased ISI). **(D)** A visualization of the interoception brain network using Brian Net Viewer (Xia et al., 2013). Edge thickness is weighted by the test statistic.

We found that TARA resulted in significant increases in connectivity compared with controls for a subnetwork of connections in the adolescent interoceptive network (NBS *p* = 0.009; test-statistic >1.35; Figure 5). We found that TARA was associated with increases in connectivity between the bilateral caudate nuclei and between the right caudate and right putamen; these connections were the strongest to improve. The right insula and the bilateral putamen also increased in connectivity strength. Other strengthened connections were between the left frontal gyri (dorsomedial, middle, and superior frontal gyri) and sensory regions including the somatosensory pre-central and post-central gyri, the middle occipital gyri, and subcortical nuclei including the caudate and putamen.

We tested whether the change in this subnetwork’s strength (sum of all connections) showed any significant correlation with the SDQ or ISI. We found that across all subjects, the interoceptive network showed a negative Pearson’s correlation with changes in the ISI (*r* = −0.23, *p* = 0.03; Figure 5C) but not for changes in the SDQ (*r* = 0.04, *p* = 0.71). Neither of the questionnaires showed a significant group-wise interaction with the subnetwork’s strength.

We tested whether TARA related to any significant SC *decreases* in this same interoception network, but this showed no significant subnetworks (*p* > 0.8).

## Discussion

In this study, we show that an innovative neuroscience-based mindfulness intervention, the Training for Awareness, Resilience, and Action (TARA), significantly changes healthy adolescent FA-weighted structural brain networks with related improvements in well-being outcomes. We found that many regions involved in interoception, emotion regulation, and mindfulness, including the putamen and insula, had significantly changed structural connectivity after TARA that related to emotional well-being and sleep quality, as measured by the SDQ Emotional Problems and ISI questionnaires, respectively. Furthermore, a whole-brain network related to adolescent interoception showed significant strengthening following TARA, and changes in this network were also related to improved sleep quality. Together, these results point to TARA’s targeted effect in adolescent cortical and subcortical networks involved in mindfulness and interoception. Finally, we demonstrated the feasibility of using secure telehealth platforms and online electronic data capture systems to fully remotely deliver to adolescents our innovative TARA intervention, which showed sufficiently high consistency, tolerability, safety, recruitment, fidelity, adherence, and retention.

Our results suggest that interoceptive brain networks may be significant to adolescent well-being and modified by mindfulness-based practices. Interoception broadly is the noticing of internal bodily sensations, which can be both overt and subconscious (Chen et al., 2021). In the practice of mindfulness, by attending carefully to internal sensations such as the breath, heart, or body posture, we directly interact with interoceptive systems (Gibson, 2019). Lesion studies have identified many significant regions involved in interoception, such as the insula, caudate, and putamen, as well as regions that are not generally considered interoceptive, such as the middle frontal gyrus and the middle temporal gyrus (Grossi et al., 2014). A recent study has shown that many of these same regions are also involved in adolescent interoception, with some notable differences in laterality, especially an underrepresentation of the right insula (Klabunde et al., 2019).

The relationship between emotion and interoception has been well documented (Barrett and Simmons, 2015; Barrett, 2017). Significant regions related to emotions are also those related to interoception, including the insula, ACC, and prefrontal cortex (Craig, 2009; King, 2019). For example, the insula has previously been shown to have increased gray matter volume related to subjective well-being (Jung et al., 2022). The present study adds to this previous study by showing a relationship between emotional well-being improvement in TARA participants and increased right insula connectivity strength. However, our findings that the right ACC node strength was related to improved well-being only in controls is against our expectations. Furthermore, the interoceptive network that strengthened in TARA cannot account for the improved emotional well-being. Since the insula’s connectivity in our study shows an association with well-being that is also not observed in the interoceptive subnetwork, this suggests that improved well-being may correspond to the insula strengthening structural connectivity to other brain regions outside of the interoceptive system. One study found that functional connectivity between the insula and the default mode network (DMN) regions is related to improved subjective well-being (Li et al., 2020), which may suggest this as a possible pathway for the insula to effect emotional improvement. On the other hand, the ACC is involved in a multitude of functions such as reward value (Shenhav et al., 2013), motor control (Paus, 2001), cognitive control (Shackman et al., 2011), and social pain (Eisenberger, 2015), and therefore, it is possible that adolescents who have improved emotional well-being outside of the TARA group may use alternative strategies more specific to ACC connectivity. The ACC in the present study was parceled with the AAL atlas, which ignores many potentially relevant subdivisions of the ACC, such as the subgenual ACC that is involved in depression (Drevets et al., 2008); these subdivisions of the ACC may differently influence the structural network strength. More studies on mindfulness interventions will be necessary to disentangle which regions and between-network connectivity can be successfully targeted by mindfulness.

Empirically, there is much overlap between regions involved in mindfulness and other types of meditation and regions related to interoception and emotion regulation. For example, mindfulness has been associated with increased gray matter density in the right insula and left inferior temporal gyrus (Hölzel et al., 2011). A recent systematic review has shown that a similar set of regions, including the caudate, putamen, ACC, insula, somatomotor cortex, and occipital cortex, is involved in both traditional sitting meditation and active-mindfulness practices, such as yoga; of which, TARA includes both (Acevedo et al., 2016). Our network-based statistic results are well aligned with this previous study, highlighting that a network of these regions undergoes significant white matter FA strengthening following an intervention, such as TARA, with both meditative- and active-mindfulness practices.

Many of the regions involved in interoception, emotion regulation, and mindfulness practice are also related to sleep regulation. In our previous clinical pilot on TARA, sleep was significantly improved at 6 months of follow-up in a sample of clinically depressed and/or anxious adolescents (Blom Henje et al., 2016). Greater sleep duration variability was related to lower white matter FA throughout the brain in a longitudinal study with adolescents (Telzer et al., 2015). It has also been shown that early adolescents with short sleep durations, defined as less than 7 h per night, exhibited reduced structural connectivity in the corticostriatal tract, which connects the caudate and putamen to the frontal cortex (Haber, 2016; Yang et al., 2023). In young men, sleep deprivation for 36 h was also related to decreased resting state functional connectivity in the caudate and putamen (Wang et al., 2021). In summary, disturbed sleep regulation is strongly associated with changes in subcortical regions and their cortical connectivity, which is in agreement with our results that improved sleep quality after TARA was related to the right putamen node strength and the strengthened interoceptive subnetwork. These findings suggest that mindfulness practices, such as TARA, improve sleep quality with implications for white matter networks in adolescents.

Our present study used the recently developed innovative TARA intervention, which was conceived with neuroscience-informed predictions about expected brain changes. As described, TARA uses a diverse suite of techniques to enact targeted changes: breath and yoga practices target sensory and interoceptive awareness; empathetic listening and compassion-oriented mindfulness practices target social and emotional brain regions; and acceptance, commitment, and mindful awareness of rewards target subcortical striatal regions involved in rewarding and craving sensations (Blom Henje et al., 2014, 2016). In TARA, mindfulness practices act synergistically with cognitive reframing and educational components to enact long-lasting changes as shown in our previous study (Blom Henje et al., 2016). However, as this study shows by the blinded and randomized controlled design, the expected target regions actually undergo network-level changes during the 12-week TARA intervention.

Together, these results indicate that TARA may be a useful intervention to support adolescent brain development to improve sleep and emotional well-being, which completely aligns with the original intention of TARA. Considering that an estimated 62.5% of individuals with mental health disorders have onset before 25 years (Solmi et al., 2022), the most important impact of this study lies in the possibility of offering a fully remotely delivered and neuroscience-based mindfulness intervention to improve mental health and create resilience among young people living across diverse urban and rural areas—including more remote areas that lack access to adequate mental health care. Such a possibility is important, given the current lack of sufficient and timely access to adequate mental health care for adolescents in need. Future larger studies are warranted to assess the efficacy, effectiveness, and real-life implementation of our fully remotely delivered TARA intervention both in community and clinical settings.

### Limitations and future directions

The results of the present study should be interpreted considering its limitations. First, although our ISI result survived a Bonferroni correction, our other findings should be cautiously interpreted in light of their exploratory nature. We note that the present study was conducted with healthy adolescents with subclinical levels of depressive symptoms. TARA was designed as an intervention for clinically depressed adolescents. A community sample as used in the current study would not be expected to show the same reductions we might observe in a clinical population. Future studies will examine whether 12 weeks of TARA would produce larger changes in depressed adolescents. Second, while to the best of our knowledge, this RCT study represents the largest fully remotely delivered mindfulness intervention using a secure HIPAA compliant telehealth platform for adolescents to date; our findings indicate that our sample size may not have been large enough to capture more subtle network changes in regional connectivity strength. For example, even though we find that the TARA group has brain changes associated with self-reported symptoms, the overall change between the two groups remained statistically the same. Third, while our study used a rigorous randomized controlled trial design, our control group was a passive waitlist group rather than an active control group. While waitlist controls are common, this also means that our ability to localize brain changes to specific mindfulness practices, rather than simply resulting from participation in an intervention, is limited. It is encouraging that our results align well with previous mindfulness research, but future studies will need to include an active control arm to assess these relationships more precisely with TARA’s mindfulness practices. Fourth, while our study demographics were moderately diverse, our sample was lacking in low-income populations and approximately half of participants were Caucasian. Therefore, future studies would benefit from including a wider range of demographics, thereby increasing the generalizability of our findings. Fifth, another limitation is that the SDQ questionnaire refers to the subject’s experience over the past 6 months and might be not sensitive enough to the temporal changes over a 12-week time period. Related to the longitudinal changes in the brain and behavior, it would also be beneficial if future studies follow and assess the study participants for a longer period of time after TARA and waitlist completion. Sixth, the limitations of the DTI methodology can reduce the accuracy of mapping white matter connections (Jones et al., 2013). Finally, a limitation of measuring changes in brain networks is that the measures can have unclear interpretability. For example, while we find that changes in node strengths are related to changes in self-reported symptoms at the group level, this cannot elucidate which connections exactly are changing. This is especially challenging since testing each individual connection is not feasible if rigorously correcting for multiple comparisons. We have included the network-based statistic as a means of enhancing the overall interpretability of our study; since the identified subnetwork only related to improved sleep quality and not to improvements in emotional problems, the question of which connections confer improved emotional well-being remains. Future studies may need to use more sophisticated network analysis strategies, as well as functional network mapping, to improve the overall interpretability.

## Conclusion

To the best of our knowledge, the current study is the largest RCT to date to examine changes in the brain in a fully remotely delivered group of mindfulness-based interventions for adolescents. Adolescence is a vulnerable neurodevelopmental phase that has significantly increased the risk for the development of key mental health disorders, such as depression, anxiety, and sleep disorders. Adolescence is also an ideal neurodevelopmental period to intervene due to a significant increase in neural plasticity and enhanced opportunity to induce greater changes in the brain networks to treat and prevent important adolescent mental health disorders, such as depression, anxiety, and sleep disturbance. This study showed that the TARA intervention has positive psychological effects with associated brain changes in adolescents. TARA improved overall sleep quality, and TARA increased structural connectivity in interoceptive brain regions and networks that related to improved sleep quality and emotional well-being. As our findings suggest that TARA affects brain regions in healthy adolescents, which are also known to be altered in adolescent depression, future studies may aim to examine the effects of TARA on clinically depressed adolescents. Since TARA and all assessments, except for the MRI scans, were fully remotely delivered using secure telehealth platforms and online electronic data capture systems, the present study demonstrates the feasibility of a fully remotely delivered TARA intervention to adolescents. In addition, we demonstrated the feasibility of utilizing fully remote recruitment methods that meet data security methods for collecting and assessing individual-level data. Finally, we proved the feasibility of conducting a randomized controlled clinical trial to investigate TARA that delivered fully remotely online as the study showed high consistency, tolerability, safety, recruitment, fidelity, adherence, and retention. Given the current lack of access to adequate mental health care for many young people who live in both urban and rural areas, there is a great need to address this critical gap in mental healthcare. The TARA intervention stands as a strong option to fill this gap and contribute to better mental health and resilience for adolescents and future generations.

## Data availability statement

The datasets presented in this article are not readily available because this study has restricted data sharing and usage; however, raw, anonymized data may be made available upon request to the corresponding author. Requests to access the datasets should be directed to OT (olga.tymofiyeva@ucsf.edu) or TY (tony.yang@ucsf.edu).

## Ethics statement

The studies involving humans were approved by University of California, San Francisco Institutional Review Board. The studies were conducted in accordance with the local legislation and institutional requirements. Written informed consent for participation in this study was provided by the participants’ legal guardians/next of kin.

## Author contributions

OT: Conceptualization, Data curation, Formal analysis, Funding acquisition, Investigation, Methodology, Project administration, Resources, Software, Supervision, Writing – original draft, Writing – review & editing. BS: Data curation, Formal analysis, Investigation, Methodology, Project administration, Software, Validation, Visualization, Writing – original draft, Writing – review & editing. TL: Data curation, Formal analysis, Investigation, Methodology, Software, Validation, Writing – original draft, Writing – review & editing. EHa: Writing – original draft, Writing – review & editing. TS: Data curation, Formal analysis, Writing – original draft, Writing – review & editing. TH: Methodology, Validation, Writing – review & editing. DG: Methodology, Validation, Writing – review & editing. AJ: Data curation, Investigation, Methodology, Project administration, Validation, Writing – review & editing. YL: Methodology, Project administration, Supervision, Writing – review & editing. TN: Writing – review & editing. EHe: Methodology, Writing – review & editing. TY: Funding acquisition, Investigation, Methodology, Project administration, Supervision, Writing – review & editing.
