# Supplementary material for: Interoceptive brain network mechanisms of mindfulness-based training in healthy adolescents
Source: Front Psychol. 2024 Aug 13;15:1410319. doi: 10.3389/fpsyg.2024.1410319 (PMC11348390; doi:10.3389/fpsyg.2024.1410319)
Supplement: Supplementary file 1 [file Data_Sheet_1.PDF]

Supplement: Interoceptive Brain Network Mechanisms of Mindfulness-Based Training in Healthy Adolescents

SUMMARY:

1. Consistency of the intervention delivery

94% of the subjects randomized to the TARA intervention participated in at least 75% of the sessions. (Go/No-Go: at least 80% of the subjects will participate in at least 75% of the sessions).

2. Tolerability of the intervention

100% of the subjects randomized to TARA had a median Child Session Rating Scale (CSRS) score of at least 20. (Go/No-Go: a median CSRS score of at least 20 in >80% of participants).

3. Safety of the intervention

There were no (0%) intervention-related adverse events (AEs). There was a total of 10 mild and one severe AEs, all of which were unrelated to the intervention. (Go/No-Go: <15% of the participants experience a grade 2 (moderate) or grade 3 (severe) adverse event related to the TARA intervention).

4. Ability to recruit subjects in the clinical study

The randomization target of 100 adolescents has been successfully achieved within the specified timeframe. All participants met entry criteria. (Go/No-Go: randomization target of 100 adolescents).

5. Ability to retain subjects in the clinical study

94% of randomized participants were retained for the primary outcome measurement at the end of the study. (Go/No-Go: retain at least 80% of randomized participants for primary outcome measurement at the end of the study regardless of adherence to the intervention).

ADDITIONAL DETAILS:

Consistency of the intervention delivery

Go/No-Go Criterion: at least 80% of the subjects will participate in at least 75% of the sessions

Attendance was recorded at each TARA session by the research personnel.
Result: In our study, 94% of the subjects who started TARA participated in at least 75% of the sessions

Tolerability of the intervention

Go/No-Go Criterion: a median Child Session Rating Scale (CSRS) score of at least 20 in >80% of participants.

The participants' acceptability of TARA was assessed weekly using the Child Session Rating Scale (CSRS) (*Campbell & Hemsley, 2009*). The CSRS is a 4-item self-assessment using a 10-cm visual analog scale, with higher scores indicating better experience. Participants rated each session in terms of how much they felt listened to (choosing on a continuous scale between "The teachers did not always listen to me" and "The teachers listened to me"), how important the content and activities were to them (choosing on a continuous scale between "What we did and talked about was not really that important to me" and "What we did and talked about were important to me"), how much they liked the session (choosing on a continuous scale between "I did not like what we did today" and "I liked what we did today"), and their overall experience (choosing on a continuous scale between "I wish we could do something different" and "I hope we do the same kind of things next time"). **Result:** 100% of the subjects randomized to TARA had a median Child Session Rating Scale (CSRS) score of at least 20.

Safety of the intervention

Go/No-Go Criterion: <15% of the participants experience a grade 2 (moderate) or grade 3 (severe) adverse event related to the TARA intervention.

Solicited adverse events in TARA participants were captured in the following manner: (1) before each TARA session participants were asked through a questionnaire: "Has there been any significant unfavorable change in your mental or physical health since last class?"; (2) if the participant answered "no" to this question, then no additional information was requested; (3) if the participant answered "yes" to this question, then the research assistant or another study staff member asked the participant for additional details regarding the participant's answer and this information was recorded in the Adverse Event form. **Result:** There were no (0%) intervention-related adverse events (AEs). There was a total of 10 mild and one serious AEs, all of which were unrelated to the intervention.

There was one serious adverse event reported during the study period; a participant experienced suicidal ideation which resulted in hospitalization. The parent of the participated stated that the cause of this event was related to family difficulties and not related to the TARA intervention. None of the reported other (non-serious) adverse events reached 5% frequency and none of them were related to the TARA intervention.

Ability to recruit subjects in the clinical study

Go/No-Go Criterion: randomization target of 100 adolescents.

Recruitment of potentially eligible participants was primarily performed in San Francisco Unified School District, serving diverse populations. **Result:** The randomization target of 100 adolescents has been achieved within the specified timeframe. All participants met entry criteria.

Ability to retain subjects in the clinical study

Go/No-Go Criterion: retain at least 80% of randomized participants for primary outcome measurement at the end of the study regardless of adherence to the intervention

A total of 94 adolescents completed the study. The drop-out rate was 6%. Thus, 94% of randomized participants were retained for the primary outcome measurement at the end of the study regardless of adherence to the intervention. Overall, 6 subjects dropped out: 1 participant left the USA and could not continue participating in the study; 2 participants failed their UCSF COVID health screening and could not be scanned at the UCSF MRI facility due to UCSF COVID prevention policy; 1 participant had a potential MRI contraindication; 1 participant had MRI claustrophobia; 1 participant had an *unrelated* serious adverse event (SAE). **Result:** 94% of randomized participants were retained for the primary outcome measurement at the end of the study regardless of adherence to the intervention.

Data completeness

Go/No-Go Criterion: primary outcome measurement for at least 80% of randomized participants

All primary outcome data (Putamen structural node strength) for the retained subjects (94% of the randomized participants) are complete. Thus, we successfully obtained the primary outcome measurement in 94% of randomized participants.
